# Targeted profiling of the serum proteome associates increased FGF-23 levels with postoperative delirium in cardiac surgical patients

**DOI:** 10.1038/s44400-026-00084-w

**Published:** 2026-04-27

**Authors:** Christopher Simon, Occam Kelly Graves, Va’Shayna E. Williams, Isabella Turco, Ariel Mueller, Miriam Trigo, Talia Collechi, Malek Mitchell, Oluwaseun Akeju, Tina B. McKay

**Affiliations:** Mass General Brigham Department of Anesthesiology, Massachusetts General Hospital, Harvard Medical School, Boston, Massachusetts USA

**Keywords:** Biomarkers, Diseases, Medical research, Neurology, Neuroscience

## Abstract

Occurrences of delirium, in the event of cardiac surgical interventions, are often confounded by antecedent metabolic states that may predispose vulnerable elders to early-onset dementia. An in-depth capture of the circulating proteome, ideally suited for discerning patients’ metabolic profiles before and after surgery, could thus be of great relevance to the delirium-dementia interface. Using a nested case-control study design, we performed targeted proteomics on serum samples collected from 19 older patients, of whom 8 (42%) experienced delirium post-surgery, and measured the levels of 183 proteins in 2 multiplex panels. Differential expression analyses identified 2 proteins associated with delirium incidence, based on a threshold of 1.5-fold difference between delirious and non-delirious inpatients. Postoperative serum fibroblast growth factor 23 (FGF-23) levels were 3.82-fold higher in patients with delirium and significantly correlated with serum neurofilament light levels. We corroborated these findings in an independent, age- and sex-matched cohort of 16 patients with similar delirium rates (38%), wherein a significant trend toward higher FGF-23 levels was observed amongst delirious patients. A technical validation of the differentially expressed FGF-23 protein via enzyme-linked immunosorbent assays further reinforced the generalizability of our results, suggesting that circulating FGF-23 may serve as a serum biomarker of delirium post-cardiac surgery.

## Introduction

Delirium, a neurocognitive disorder of altered consciousness and fluctuating manifestations, bears a multi-factorial etiology that accounts for its rapid onset and adverse, albeit transient, sequelae in elderly surgical patients^1^. Delirial episodes post-surgery, though reversible, may thus appear to reflect a prodrome that precedes neurodegeneration, as evidenced by its robust associations with markers of neuroaxonal damage, namely neurofilament light (NfL), tau, and TAR DNA-binding protein 43^2–5^. Amongst the latter, circulating NfL and tau levels, in particular, were seemingly driven by surgical severity, considering that their trajectories trended towards an immune-responsive state, with profound upregulation of inflammatory cytokines such as interleukin (IL)-6, IL-8, and IL-10^6,7^. Although this substantial correlation was shown, at least in part, to foster delirium vulnerability in distinct subsets of surgical populations, the reciprocal influence of such dynamics has yet to be convincingly paralleled in the cardiac surgical setting^8^.

Despite the inception of an overt neuroinflammatory phenotype, presumably due to sustained surgical trauma, co-existing metabolic correlates, by proxy, exhibited changes that might have precipitated the occurrence of delirium after cardiac surgery^9^. Among cardiac surgical patients, deficiencies in 25-hydroxy vitamin D levels prior to surgery^10^ and elevations in fibroblast growth factor (FGF)-21 levels, amidst a parallel surge in IL-6, following surgery^9^, correlated significantly with delirious outcomes. While some of these changes may exemplify a peripheral etiology that is perpetuated by surgical stress, it is uncertain whether this accounts for the overall delirial phenotype, since antecedent metabolic states did not always coincide with the onset of delirium. Though significant, post-surgical increases in serum FGF-23 levels, albeit an IL-6 surge, displayed a lack of correlation with delirious symptoms, prompting speculation that a neuropathological threshold exists and must be surpassed^9^. Pre-surgical vitamin D deficits, likewise, were neither discriminative nor diagnostic of post-cardiac surgery delirium in certain clinical cohorts^11^, further implying an unmet need to capture the circulating determinants that drive the transition of such thresholds for symptom emergence. Indeed, the co-occurrence of immune and vascular events following any surgical intervention may exacerbate underlying metabolic vulnerabilities and potentially contribute to delirium’s initial symptomatology^12,13^. With the advent of high-throughput proteomic platforms^14^, such as proximity extension assays (PEAs), a targeted analysis of the circulating proteome could provide causal insights into its perioperative dynamics.

Herein, we measured the abundance of 183 serum proteins by utilizing PEA technology across 2 multiplex panels (Cardiovascular II and Immune Response) in two independent cohorts. The discovery cohort comprised patients with overnight hospital admissions before cardiac surgery^15^, while the validation cohort included patients with same-day surgical admissions^16^. Subsequently, a technical validation of the PEA results was performed via enzyme-linked immunosorbent assays in a third cohort of patients^17^. We hypothesized that patients with distinct serum metabolic signatures are more predisposed to postoperative delirium after cardiac surgery.

## Results

### Cohort characteristics

For our primary analysis, 19 inpatients scheduled for cardiac surgery with preoperative overnight stays in the hospital were evaluated for differentially expressed serum proteins using PEAs. 8 out of 19 patients (42%) experienced postoperative delirium within the first 3 days after surgery (Table 1). To verify the findings in an independent cohort, validation cohort 1 with a similar delirium proportion (6/16 = 38%) was analyzed. A technical validation of the PEA results obtained from the abovementioned cohorts was conducted in validation cohort 2 (9/24 = 38% delirium).

**Table 1:** Cohort characteristics for three independent cohorts

	Primary cohort		Validation cohort 1		Validation cohort 2	
	No delirium (*n* = 11)	Delirium (*n* = 8)	No delirium (*n* = 10)	Delirium (*n* = 6)	No delirium (*n* = 15)	Delirium (*n* = 9)
Age (years)	70 [68, 76]	73 [67, 75]	70 [62, 76]	70 [68, 72]	73 [66, 76]	72 [71, 76]
Male sex	8 (73)	5 (62)	7 (70)	3 (50)	11 (73)	6 (67)
Kidney disease	2 (18)	2 (25)	1 (10)	0 (0)	2 (13)	1 (11)
T-MoCA (baseline)	18 [16, 19]	18 [15, 19]	19 [18, 20]	19 [17, 21]	19 [18, 20]	18 [15, 21]
CAM-S (postop)	4 [2, 4]	8 [6, 10]^a^	2 [2, 3]	7 [6, 7]	3 [2, 3]^b^	9 [7, 9]
Bypass time (minutes)	146 [94, 210]	116 [100, 152]	92 [84, 120]	156 [122, 176]	177 [118, 197]	192 [148, 268]
Postoperative hospitalization (days)	6 [5, 8]	8 [7, 15]	5 [5, 6]	6 [5, 8]	7 [5, 8]	9 [7, 12]^b^

Data presented as median [interquartile range] or frequency (percent) depending on the variable type.

*T-MoCA* Telephone Montreal Cognitive Assessment, *CAM-S* Confusion Assessment Method Severity.

^a^2 values missing.

^b^1 value missing.

Across all cohorts, patients with delirium had similar age distribution, sex proportion, low kidney disease incidence, and baseline cognitive function to those without. Postoperatively, the confusion assessment method-severity (CAM-S) scores were consistently higher in delirious patients (median difference [95% confidence interval, CI] = 4 points [2–6], *p* = 0.0052; 4 points [3–5], *p* = 0.0013; and 6 points [4–7], *p* < 0.0001 in the primary, validation 1, and validation 2 cohorts respectively). In validation cohort 1, bypass time was significantly longer in the delirium group (48 minutes [1–91], *p* = 0.0420), whereas in other cohorts, similar bypass durations were displayed between groups. Although postoperative hospitalization trended toward longer stays for delirious patients, these differences were not significant in any of the cohorts.

### Preoperative to postoperative changes

**Fig. 1:**
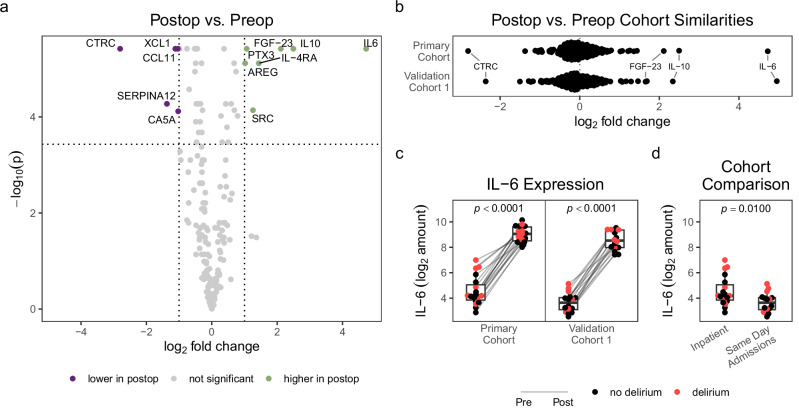
Proteomic changes following cardiac surgery highlight increases in IL-6. **a** Volcano plot representing the log_2_-fold change comparing preoperative and postoperative groups. Dotted lines indicate significance cutoffs of *p*_adj_ < 0.05 and fold change < 1/2-fold or > 2-fold. **b** Change in protein levels from preoperative to postoperative timepoints across all screened proteins, with the proteins of interest labeled. **c** Changes in IL-6 protein expression per patient. *P*-values are calculated from the Wilcoxon signed-rank test. **d** Baseline differences in IL-6 between inpatient (primary) and same-day admission (validation 1) cohorts. *P*-value is calculated from the Mann-Whitney *U* test.

To identify potential associations between the serum proteome and surgical timepoints, primary samples drawn before surgery and on postoperative day 1 were analyzed using the PEA. Across all screened proteins, inflammatory markers, as expected, had the largest increases. IL-6, in particular, had the largest increase post-surgery (26.4-fold [95%CI = 14.3−34.3], *p*_adj_ = 0.0007), followed by IL-10 (5.6-fold [3.7−9.0], *p*_adj_ = 0.0007) and FGF-23 (4.3-fold [2.7−6.8], *p*_adj_ = 0.0007) (Fig. 1a). A similar trend was observed when the same analysis was performed in validation cohort 1 (Fig. 1b, Supplementary Fig. 1a). In both cohorts, every patient’s IL-6 level increased after surgery (Fig. 1c). Intriguingly, baseline levels of IL-6 also differed between cohorts, with the primary cohort’s inpatient subjects having higher IL-6 than validation cohort 1’s same-day admission subjects (median difference = 74%, [9−155%], *p* = 0.0100, Fig. 1d).

### Delirium versus No delirium differences

**Fig. 2:**
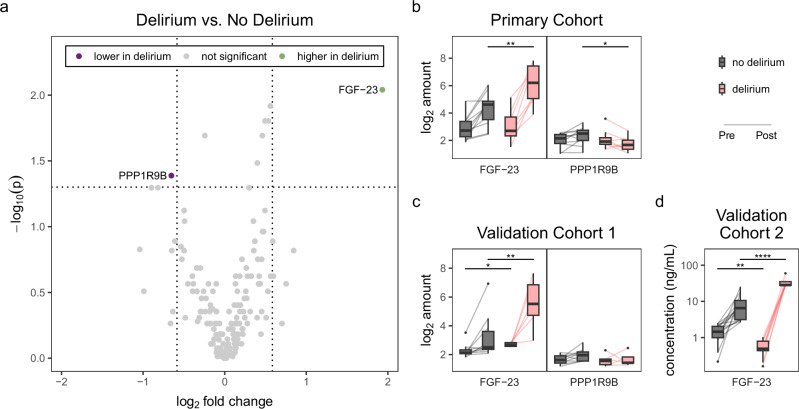
Proteomic comparisons between delirium and no delirium patients reveal differences in FGF-23. **a** Volcano plot representing the log_2_-fold change at postoperative day 1 comparing the delirium and no delirium groups. Dotted lines indicate significance cutoffs of *p* < 0.05 and fold change < 2/3-fold or > 3/2-fold. **b** Relative protein concentrations of the 2 proteins with differential regulation in the delirium group. **c** An independent validation study of the relative protein concentrations of the 2 proteins identified in the primary cohort. **d** A second independent validation study of FGF-23 concentrations using ELISAs. Mann-Whitney *U* *p < 0.05, **p < 0.01, ****p < 0.0001.

Of all 183 proteins screened using the PEA, we found that one protein, FGF-23, had higher postoperative levels in delirious patients (3.82-fold [95% CI = 1.26−8.13], *p* = 0.0091), and one protein, PPP1R9B, had lower postoperative levels (0.64-fold [0.42−0.99], *p* = 0.0409, Fig. 2a, b). None of these were significant following multiple comparison adjustments. Subsequent analysis of these two proteins in validation cohort 1 revealed that postoperative FGF-23 levels remained significantly elevated in the delirium group (6.38-fold [1.52−25.68], *p* = 0.0075, *p*_adj_ = 0.0150, Fig. 2c, Supplementary Fig. 1b). When the postoperative results from both cohorts were combined, FGF-23 was the only protein that was significantly differentially expressed after multiple comparison adjustment (Supplementary Fig. 2b). Further investigations in validation cohort 2, via ELISAs, corroborated the higher postoperative levels of FGF-23 in delirious patients (4.36-fold [2.66−9.40], *p* < 0.0001, Fig. 2d). Considering these differences, determinants of FGF-23 homeostasis were then quantified to explore its metabolic feedback. Notably, vitamin D, klotho, and phosphate concentrations showed no alterations between the delirium and no delirium groups. Moreover, none of the serum levels were shown to be significantly correlated with serum FGF-23 (Supplementary Fig. 3). Preoperatively, no proteins were consistently altered between the delirium and no delirium groups in all cohorts (Supplementary Fig. 2a, 4).

**Fig. 3:**
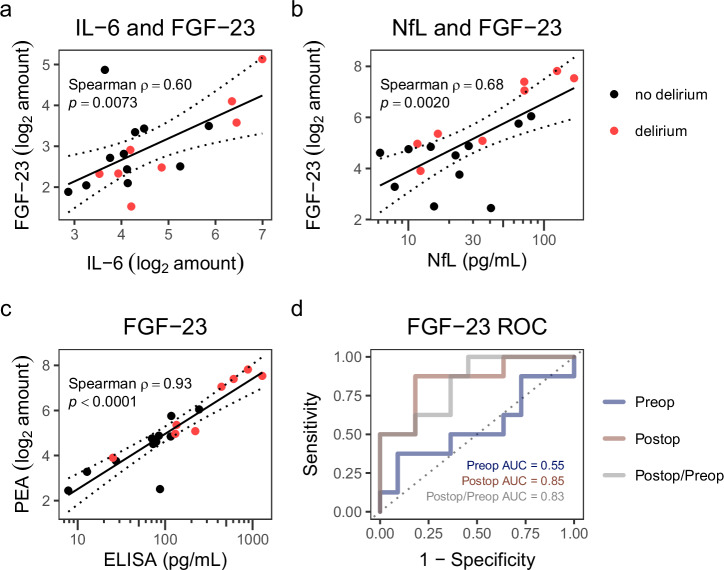
Correlations of FGF-23 and association with postoperative delirium. **a** Baseline comparison between IL-6 and FGF-23 levels. The solid line indicates the least squares regression, and the dotted lines show the 95% CI. **b** Correlation analysis of NfL and FGF-23 serum levels on postoperative day 1. **c** Correlation analysis between PEA and ELISA measurements of postoperative FGF-23 serum levels. **d** Receiver operative characteristic (ROC) curves and the area under the curve (AUC) values for FGF-23 levels at preoperative, postoperative day 1, and perioperative change, as discriminators of delirium.

At baseline, IL-6 correlated with serum FGF-23 levels (Spearman ρ = 0.60 [0.21−0.83], *p* = 0.0073, Fig. 3a); a correlation that was not maintained on postoperative day 1 (Supplementary Fig. 5a). Motivated by this finding, we next compared postoperative serum NfL and FGF-23 levels and found a significant positive correlation (Spearman ρ = 0.68 [0.32–0.86], *p* = 0.0020, Fig. 3b, Supplementary Fig. 5b), suggesting the concomitant presence of neuroaxonal damage. In support of the replicability of our findings, we identified a strong correlation between the serum levels of FGF-23 measured by both PEAs and ELISAs (Spearman ρ = 0.93 [0.82−0.97], *p* < 0.0001, Fig. 3c). Furthermore, the postoperative FGF-23 measurements discriminated between the no delirium and delirium groups effectively, with an optimal sensitivity-specificity combination at 0.88 sensitivity and 0.82 specificity, and an area under the receiver operating characteristic curve of 0.85 (*p* = 0.0091, Fig. 3d).

## Discussion

In this study, we leveraged the proximity extension assay platform and revealed that elevated serum fibroblast growth factor-23 (FGF-23) levels are associated with post-cardiac surgery delirium in elderly patients. This trend was observed despite a lack of correlation with serum 25-hydroxy vitamin D, klotho, and phosphate levels, which, along with acute hospitalization, did not coincide with the occurrence of delirium in our surgical inpatient cohort. Subsequent analysis of two independent validation cohorts, comprising same-day surgical admissions, further corroborated the FGF-23 increase in delirious patients.

FGF-23, a bone-derived metabolic correlate of renal and cardiovascular injury, has been reported to govern the homeostasis of vitamin D and phosphate by means of its obligate co-receptor Klotho^18,19^. Among these metabolites, the endogenous expression of Klotho on human vasculature, in particular, imparts vascular resilience by rendering them responsive to FGF-23^20,21^. When the circulating milieu exhibits an imbalance towards this metabolic feedback, however, sustained elevations of serum FGF-23 levels, following cardiac surgical interventions, may lead to aberrant vascular remodeling^22^. The uncoupling of FGF-23 from its feedback control, likely attributable to deteriorating renal function that accompanies ageing, could be seen along with perivascular or peri-surgical inflammation^22^, and is known to precipitate cognitive dysfunctions^23^. Considering that a more accentuated serum FGF-23 increase was reported to correlate with perioperative kidney function within the first few days after cardiac surgery^24–27^, the development of delirium post-surgery might thus be an inevitable consequence of compromised renal vasculature. Despite this, the extent to which post-cardiac surgery increases in serum FGF-23 levels, observed across both our delirious inpatient and same-day admission cohorts, is causally associated with delirium onset, remains unclear. Beyond renal excretion dynamics, our post-surgical FGF-23 measurements and delirium assessments were carried out in close temporal proximity alongside stresses of surgery, suggesting that high FGF-23 levels could also be a general reflection of systemic or vascular vulnerability. Whether elevated circulating FGF-23 in the perioperative phase reflects susceptibility or an acute delirious event, incorporating multiple precisely-defined sampling times that coincide with delirium symptomatology would be of utmost importance for unravelling FGF-23’s true diagnostic value in future clinical studies.

While our findings support the notion that FGF-23 is increased in a manner that is not driven by its feedback loop, the intricate interplay underlying such altered patterns of serum dynamics, which goes beyond any surgical intervention, remains an area of considerable ambiguity. Though it mirrored the high serum trajectories of IL-6 at baseline, prolonged hospital stays were less likely to be a perpetuating factor, since the primary cohort’s inpatient subjects were not hospitalized due to prior illnesses. This concomitance was not maintained on postoperative day 1, and FGF-23’s post-surgical shift in correlation with serum NfL levels instead signifies a predisposition towards neuroaxonal damage and subsequent degeneration in delirious patients. It is uncertain to what extent FGF-23’s increases are of peripheral or central origin in the systemic vasculature, but if causal, there seems to be a threshold of pathology above which the onset of delirium appears and below which it does not^9^. In older patients who are acutely delirious following cardiac surgery, the risk of developing dementia within 5 years is substantially higher^28^, thus warranting consideration of long-term follow-up. Given the small sample size, which runs the risk of overfitting, replicating our findings in larger, more diverse populations and integrating FGF-23 into multi-biomarker risk scores that capture the delirium-dementia continuum may provide a more holistic view beyond its peri-surgical trajectory. Nevertheless, confounding variables such as age, sex, bypass time, perioperative complications, vascular comorbidities, and kidney function should first be accounted for, not only to reconcile the seemingly conflicting influence of FGF-23 on baseline susceptibility, but also to strengthen its stand-alone utility as a diagnostic marker for delirium post-cardiac surgery.

## Methods

### Study design

All experiments were performed in accordance with guidelines and regulations approved by the MGB Institutional Review Board (IRB Protocol #: 2018P000480, 20168000742, 2022P000445). This study analyzed the preoperative and postoperative levels of serum proteins in three cardiac surgery cohorts using a nested, case-control study design: a primary cohort with 19 patients (*n* = 11 no delirium and *n* = 8 delirium, Institutional Review Board, IRB protocol #: 2018P000480)^15^, a validation cohort 1 with 16 patients (*n* = 10 no delirium, *n* = 6 delirium, IRB protocol #: 20168000742)^16^ matched to the primary cohort by age, sex, and baseline telephone Montreal Cognitive Assessment (T-MoCA) scores, and a validation cohort 2 with 24 patients (*n* = 15 no delirium, *n* = 9 delirium, IRB protocol #: 2022P000445)^17^. We have previously published the analyses of serum tau levels in the primary and validation cohort 1^4,5^, of serum TDP-43 levels in validation cohort 2^2^, of serum MAP2 levels in the primary and validation cohort 2^29^, and of proximity extension assays in a separate nested case-control study of patients enrolled in the parent clinical study for validation cohort 1^9^. The primary inclusion and exclusion criteria have been described in the parent studies^15–17^, and included adults aged ≥ 60 years scheduled for cardiac surgery at Massachusetts General Hospital (MGH). Primary exclusion criteria excluded patients with blindness, deafness, or the inability to speak English and provide informed consent. All studies were conducted in accordance with the Declaration of Helsinki, and written informed consent was obtained prior to enrollment, in accordance with federal and institutional guidelines. Baseline T-MoCA was performed preoperatively^30^. The primary outcome of postoperative delirium was assessed twice daily by trained clinical research staff for three days following surgery, using the same short- and long-form Confusion Assessment Method (CAM) in each cohort^31,32^.

### Serum isolation

Blood was collected before surgery and on postoperative day 1, and drawn into serum collecting tubes with clot activators (BD Vacutainer, BD, Franklin Lakes, NJ). Samples were then allowed to clot at room temperature (RT) for 30 min and centrifuged at 1000−2000 × g for 30 min at 22 °C to pellet insoluble components. The serum was collected and aliquoted into cryovials for downstream analysis (stored at −20 °C for ≥ 24 hours, followed by transfer to −80 °C).

### Targeted proximity extension assays

Serum samples were first thawed on ice and gently mixed by trituration. 40 μL of each sample was then transferred to a 96-well plate, sealed, placed on dry ice, and sent to Olink Analysis Services for protein analysis (Olink Proteomics, Watertown, MA). Proximity extension assays (PEAs) for the Target 96 Cardiovascular II (v.5006) and Target 96 Immune Response (v.3203) panels were performed by the core facility. In short, samples were incubated with selected antibody-bound oligonucleotide pairs specific to the antigens targeted within each panel. This was followed by the addition of DNA polymerase and corresponding primers, and finally, amplification and analysis by real-time polymerase chain reaction^33^. Protein levels were reported as an arbitrary normalized protein expression (NPX) value in log_2_ units. The intra-assay % coefficient of variation (% CV) for the Cardiovascular II and Immune Response panels was reported as 3% and 5%, respectively. All proteins (92/92) on the Cardiovascular II panel were detected in >50% of samples across both cohorts, whereas 92% (85/92) and 91% (84/92) of proteins on the Immune Response panel were detected in >50% of samples in the primary and validation 1 cohorts. Given that one protein was represented on both panels, a total of 176 and 175 proteins were analyzed in the primary and validation 1 cohorts. For each panel, all samples were assayed on the same plate and normalized using the inter-plate controls (IPCs).

### Enzyme-linked immunosorbent assays

Preoperative and postoperative serum levels of the following factors or proteins were determined by ELISA according to the manufacturers’ protocols: Vitamin D (Total 25-OH Vitamin D ELISA Kit, cat. # 80987; Crystal Chem, Elk Grove Village, IL, USA), FGF-23 (Human FGF-23 ELISA Kit, ELH-FGF23; RayBio, Norcross, GA, USA), Klotho (Human Klotho DuoSet ELISA, DY5334-05; R&D Systems, Minneapolis, MN, USA), and IL-6 (Human IL-6 ELISA Kit, KAC1261; Life Technologies, Frederick, MD, USA). Briefly, serum samples were diluted 2- to 4-fold (for FGF-23 and IL-6 assays) or analyzed undiluted (for Vitamin D and Klotho assays), and incubated for 1−2.5 hours at RT or overnight at 4 °C with antibody-coated plates. Secondary antibodies and conjugates were then incubated with samples for 0.5−2 hours at RT, followed by the addition of the substrate (15 min incubation at RT) and stop solution. Absorbance measurements at 450 nm were taken using a spectrophotometer (SpectraMax Plus 384; Molecular Devices, San Jose, CA, USA). Unknown concentrations were calculated by interpolation from the standard curves.

### Serum phosphate levels

Phosphate levels were measured using a colorimetric phosphate assay kit according to the manufacturer’s protocol (ab65622; Abcam, Cambridge, MA, USA). Briefly, serum samples were diluted 200- to 400-fold, incubated with malachite green phosphate reagents for 30 min at RT, and absorbances read at 650 nm. Sample concentrations were interpolated based on a standard curve generated by solutions with known phosphate concentrations of monopotassium phosphate (KH_2_PO_4_, CAS Number: 7778-77-0; Sigma-Aldrich, St. Louis, MO, USA).

### Single-molecule immunoassay

NfL serum levels were measured using the single-molecule array (Simoa) platform according to the manufacturer’s instructions (Quanterix, Billerica, MA, USA). Briefly, 25 µL of serum and standards of known concentrations were incubated with capture and detector antibodies while shaking, followed by washing and incubation with SβG reagents. The plates were re-washed, dried, and finally analyzed on the SR-X instrument (Quanterix). Absolute concentrations were determined by interpolation from a standard curve.

### Total protein concentration

Serum proteins were quantified using a bicinchoninic acid (BCA) assay (Pierce BCA Protein Assay Kit; Thermo Fisher Scientific, Rockford, IL, USA). Samples were diluted 100-fold, incubated with BCA reagents in a 1:8 ratio for 30 minutes at RT, and the absorbance was measured at 562 nm on a SpectraMax Plus 384 spectrophotometer (Molecular Devices, San Jose, CA, USA).

### Statistical analysis

All statistical analysis was performed using GraphPad Prism (Version 10.0.0 for Windows, GraphPad Software, Boston, Massachusetts, USA) and R Studio (R Version 4.4.3, RStudio, Inc., Boston, Massachusetts, USA). Normality of values was evaluated using the Shapiro-Wilk test, and non-parametric tests were used to determine statistical significance between groups. Differences between the delirium and no delirium groups were evaluated using multiple Mann-Whitney *U* tests. A Wilcoxon matched-pairs signed-rank test was applied to compare preoperative and postoperative values. Spearman correlations were used to determine associations between different biomarkers and biomarker levels detected by different analytical approaches (PEAs versus ELISAs). The Holm correction was used for multiple-comparison *p*-value adjustments (*p*_adj_) when screening multiple proteins. Exploratory analyses in the primary cohort have unadjusted *p*-values (*p*) listed. A *p*-value of *p* < 0.05 was considered statistically significant.

## Supplementary information

Supplementary information

## Data Availability

The datasets generated and/or analyzed during the current study are not publicly available due to patient data privacy but are available from the corresponding author on reasonable request.
