# Supplementary material for: Targeted profiling of the serum proteome associates increased FGF-23 levels with postoperative delirium in cardiac surgical patients
Source: NPJ Dement. 2026 Apr 27;2(1):31. doi: 10.1038/s44400-026-00084-w (PMC13121006; doi:10.1038/s44400-026-00084-w)
Supplement: Supplementary file 1 — Supplementary information [file 44400_2026_84_MOESM1_ESM.pdf]

**Supplementary materials:**

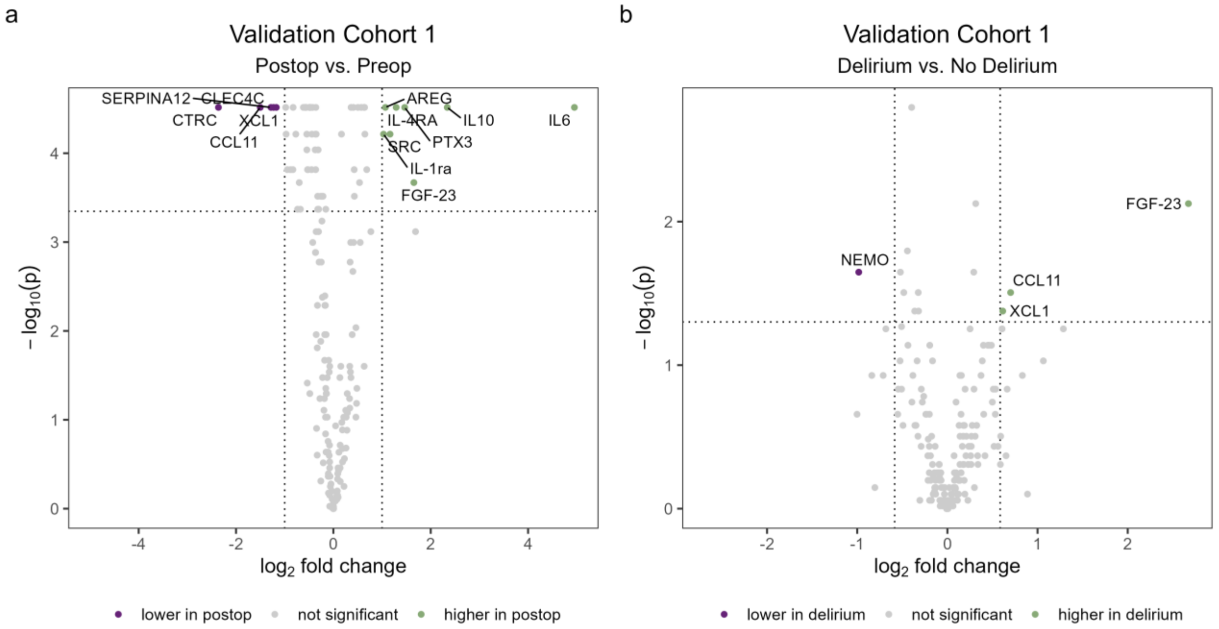


**Supplemental Figure 1. Protein differences in validation cohort 1.** (**a**) Change in protein levels from preoperative to postoperative timepoints across all screened proteins. Dotted lines indicate significance cutoffs of $p_{adj} < 0.05$ and fold change < 1/2-fold or > 2-fold. (**b**) Postoperative protein differences between delirium and no delirium. Dotted lines indicate significance cutoffs of $p < 0.05$ and fold change < 2/3-fold or > 3/2-fold.

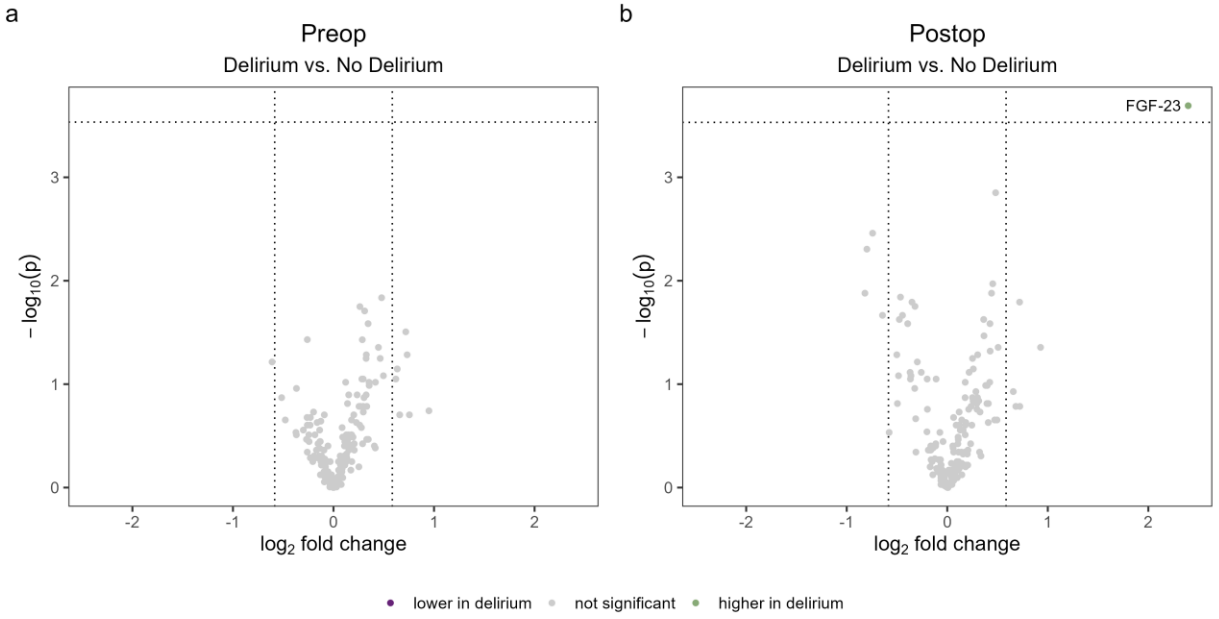


**Supplemental Figure 2. Protein differences after combining the primary cohort and validation cohort 1 (n = 21 no delirium, n = 14 delirium).** (**a**) Preoperative differences between delirium and no delirium. (**b**) Postoperative differences between delirium and no delirium. Dotted lines indicate significance cutoffs of $p_{adj}$ < 0.05 and fold change < 2/3-fold or > 3/2-fold.

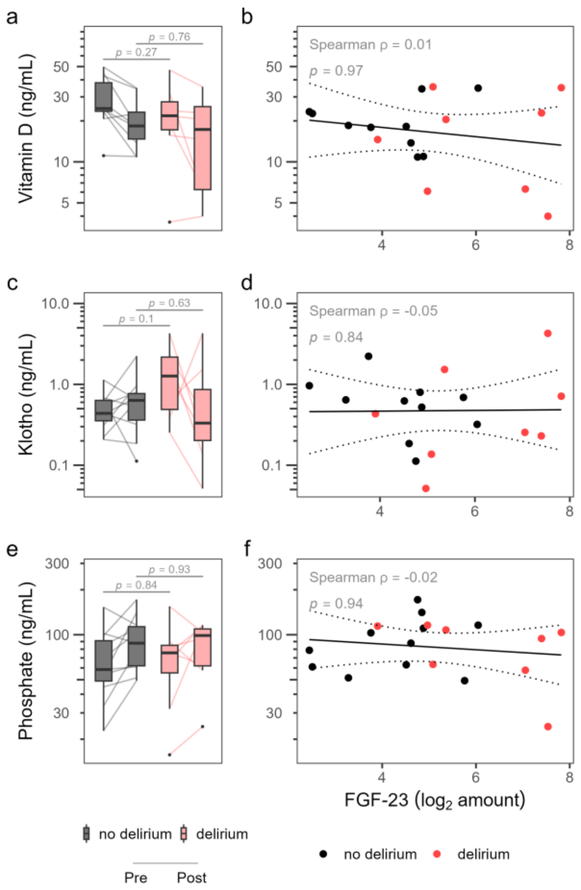


**Supplemental Figure 3. Serum concentration of FGF-23-related markers.** (**a**) The total 25-hydroxy vitamin D concentration was compared between delirium and no delirium patients across timepoints. P-value indicates the significance of the Wilcoxon rank-sum comparison between delirium and no delirium. (**b**) The total 25-hydroxy vitamin D concentration was compared to the postoperative FGF-23 concentration. The solid line indicates the least squares regression, and the dotted lines show the 95% CI. (**c-d**) Klotho concentration (**c**) was compared between delirium and no delirium patients across timepoints, and (**d**) compared to postoperative FGF-23 concentration. (**e-f**) Monopotassium phosphate concentration (**e**) was compared between delirium and no delirium patients across timepoints, and (**f**) compared to postoperative FGF-23 concentration.

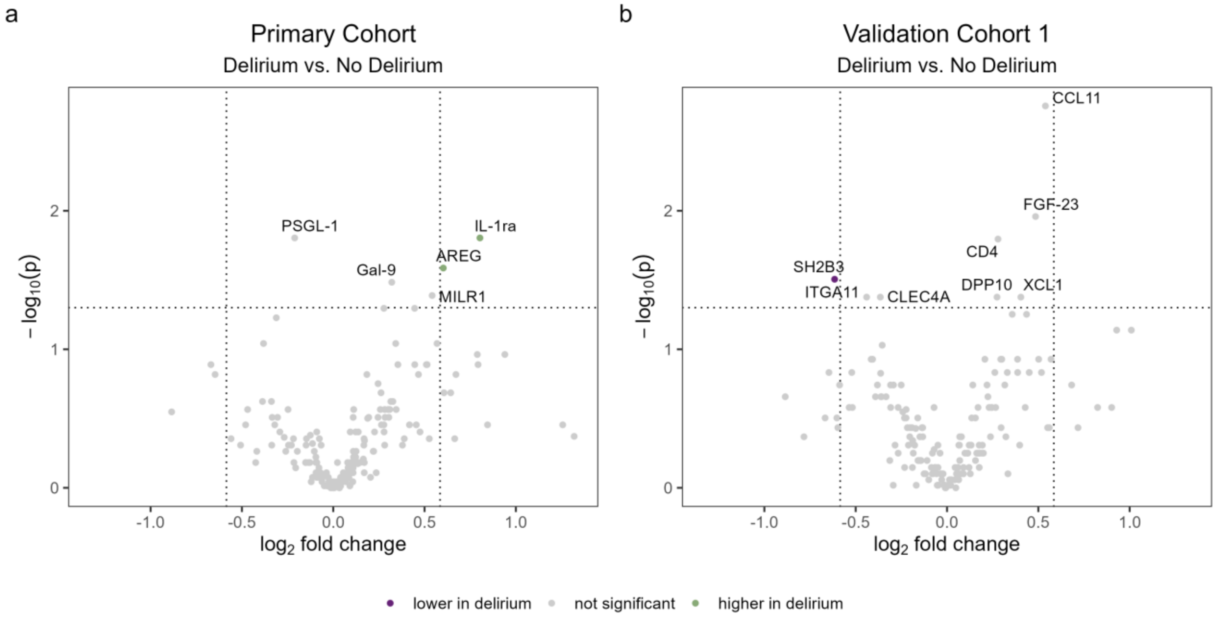


**Supplemental Figure 4. Preoperative protein differences between the delirium and no delirium groups.** Comparisons are shown for (**a**) the primary cohort (n = 11 no delirium, n = 8 delirium) and (**b**) validation cohort 1 (n = 10 delirium, n = 6 no delirium) for all 183 analyzed proteins. Dotted lines indicate significance cutoffs of $p < 0.05$ and fold change < 2/3-fold or > 3/2-fold.

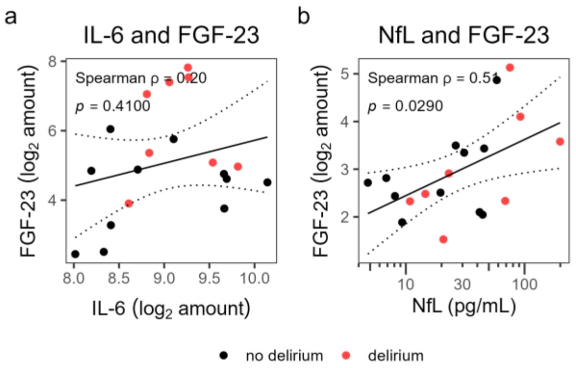


**Supplemental Figure 5. Correlations of FGF-23.** (**a**) Correlation analysis of IL-6 and FGF-23 levels on postoperative day 1. The solid line indicates the least squares regression, and the dotted lines show the 95% CI. (**b**) Baseline comparison between NfL and FGF-23 serum levels.
